# Unusual, Radical Treatment of Type A Aortic Dissection: A Case Report

**DOI:** 10.1055/s-0040-1714090

**Published:** 2020-12-11

**Authors:** Rossella M. Benvenga, Michele Bellino, Generoso Mastrogiovanni, Donato Triggiani, Rodolfo Citro, Paolo Masiello, Gennaro Galasso, Federico Piscione, Severino Iesu

**Affiliations:** 1 Department of Medicine, Surgery and Dentistry, University of Salerno, Salerno, Italy; 2 Department of Heart, University Hospital of Salerno, Salerno, Italy

**Keywords:** aneurysm, dissection, open surgical management, Florida sleeve, frozen elephant trunk

## Abstract

Type A aortic dissection, according to Stanford classification, is a surgical emergency with high morbidity and carries 56% of in-hospital mortality when surgical intervention is not performed. The surgical mortality at 30 days is 10 to 20%. The therapeutic goals are to replace the diseased ascending aorta and to treat or to monitor the distal aortic patent false lumen. When the dissection involves the aortic root and the architecture of aortic valve is normal, the surgical techniques used could be multiple: reinforce the aortic root and spare the native aortic valve or replace the aortic valve and the aortic root. The Florida sleeve technique has been developed to treat the aortic aneurysm, sparing the aortic valve in patients with connective tissue disease. Some case reports have described the use of this technique to treat an acute aortic dissection. In the following case, we present a single stage repair of the ascending aorta, aortic arch, and proximal intrathoracic aorta in a patient with Type A aortic dissection through the contemporaneous use of two techniques: Florida sleeve and Vascutek “Thoraflex” hybrid prosthesis. The use of these two techniques allows the repair/replacement of the proximal intrathoracic aorta, the sparing of the native aortic valve, the employment of a hybrid prosthesis to replace the supraortic vessels, and the creation of a descending aortic landing zone for later, distal intervention.

## Introduction

Type A aortic dissection is a catastrophic surgical emergency with well-known high morbidity and mortality, despite constant improvements in its management. 1 Important tools of the therapy are (1) replacement of diseased aorta in various amounts and (2) follow-up and treatment of the distal aortic patent false lumen. Use of the frozen elephant trunk technique is very well described in the setting of Type A aortic dissection for the repair of aortic arch and the proximal intrathoracic aorta. It is under debate which surgical technique to use when there is aortic root involvement and the aortic valve structure is preserved. The following case report demonstrates a radical approach to Type A aortic dissection in one single stage: sparing of the aortic valve, reinforcement of the aortic root, and total replacement of the ascending aorta, aortic arch, and proximal descending-thoracic aorta.

## Case Presentation

**Fig. 1:**
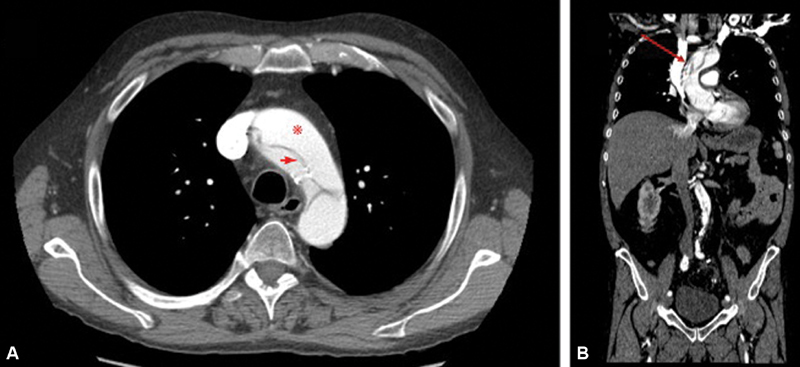
Contrast-enhanced computed tomography. ( **A** ) Axial section of the upper thorax shows the dissection flap (shown by red arrow) in the aortic arch. The true lumen is anterior (shown by red asterisks) and the false lumen is posterior; ( **B** ) coronal section of thorax and abdomen shows the Type A aortic dissection involving the ascending and abdominal aorta and proximal segment of the aortic arch, with an image of spiral intimal flap and “cobweb sign” of the false lumen (shown by red arrow).

**Fig. 2:**
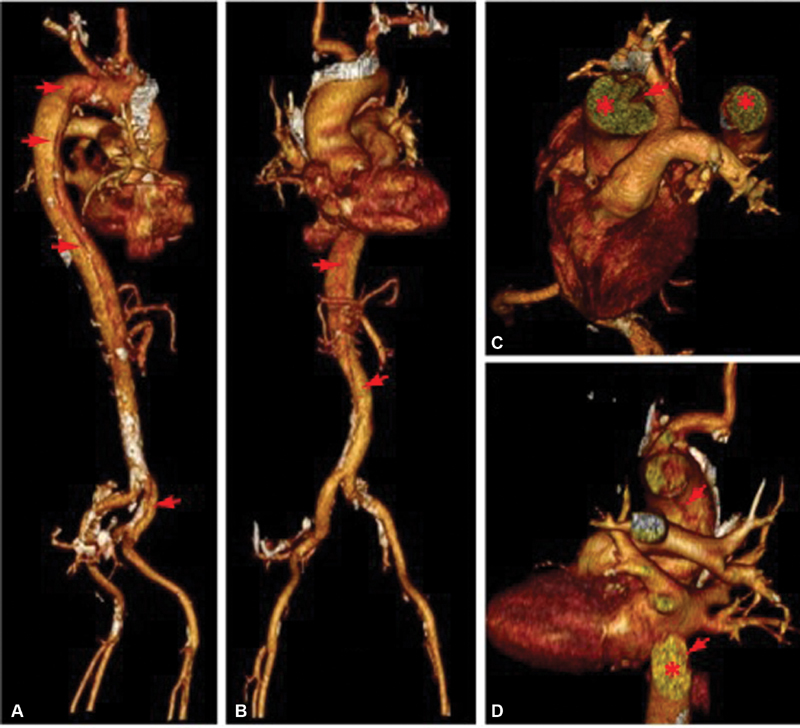
( **A, B** ) Volume rendering of entire aorta with the image of spiral intimal flap (shown by red arrows) dividing the true and false lumens and running parallel to the longitudinal axis of aorta ( **C, D** ) Volume rendering of the intrathoracic aorta; ( **C** ) posterior view with spiral intimal flap, showing the true and false lumen in the distal aortic arch; ( **D** ) axial section showing the true and false lumens in the ascending and descending aorta. The spiral intimal flap is shown by red arrows. The true lumen is shown by red asterisks.

A 60-year-old Caucasian male, nondiabetic, smoker with past medical history of hypertension was admitted to a peripheral hospital after an episode of a transient loss of consciousness, with a suspected diagnosis of alcoholic hangover. The patient reported chest pain. On physical examination, he was cold, and sweaty, with a grade-II diastolic murmur heard best at the left middle sternal border. Blood pressure was140/60 mm Hg and heart rate was 90/min. A 12-lead electrocardiogram (ECG) showed normal sinus rhytm and inverted T waves in inferior leads. Blood examinations revealed a mild increase of high sensitive troponin (68 ng/dL; normal range <40 ng/dL). The transthoracic bidimensional echocardiography (TTE) revealed severe aortic regurgitation, a 5-cm ascending aortic aneurysm and mild pericardial effusion. Computed tomography (CT) angiograph demonstrated Type A aortic dissection, extending upward to the supra-aortic vessels, and distally to the common iliac arteries, with partial occlusion of right internal carotid artery ( Figs. 1 and 2 ). The patient was immediately transferred to our institution for emergent cardiac surgery.

Operative technique: full monitoring was established, with ECG, bilateral radial artery cannulation, and central venous line insertion. After median sternotomy and a small inferior cervicotomy along the anterior border of sternocleidomastoid muscle, the right intrathoracic subclavian artery was prepared and cannulated, with interposition of an 8-mm prosthesis. The right atrium was cannulated with a two-stage cannula. Cardiopulmonary bypass (CPB) was instituted and systemic cooling started. At 30°C, the ascending aorta was clamped and crystalloid cardioplegia was given. Under cardioplegic arrest, the dissected ascending aorta was transected and excised, the aortic root was skeletonized completely, and circumferentially mobilized down to the aortic annulus level. Due to dilatation of Valsava sinus without involvement in the dissection and the preserved aortic valve structure, it was decided to repair the aortic root and the ascending aorta using the Florida sleeve aortic root technique with a bio-Valsalva prosthesis (Vascutek Gelweave Valsalva Graft).

Six Ethibond 3–0 U-stiches were passed at the annulus level (nadir + commissure) and the bio-Valsalva prosthesis was affixed. The aortic root and the aortic valve were pulled in and the sinotubular junction was sutured to the prosthesis. The sizing of bio-Valsalva's prosthesis was chosen considering dimension and form of native aortic root to avoid further deformation or dilatation and to preserve ventriculoaortic junction anatomy, the aortic leaflets attachment and the sinotubular junction, and the so-called functional aortic annulus (geometric approach). We have created specific bottom on the prothesis to accept the coronary artery ostia and sutured the prosthesis to the sinus above the coronary ostia to avoid its following avulsion. The coronary arteries were not involved in the dissection.

Thereafter the left common carotid and left subclavian arteries (via an interposed graft) were cannulated and the brachiocephalic trunk was clamped, so that total antegrade cerebral perfusion was obtained.

The aortic arch was completely resected. The Thoraflex system was gently bent and then advanced into the descending thoracic aorta. Once the stent graft had been deployed, the distal anastomosis was performed. The fourth branch of the graft was cannulated, the prosthesis clamped, and lower body reperfusion and partial rewarming initiated. Sequential reattachment of left subclavian and left common carotid arteries to the side branches of the Thoraflex was performed. The dissected ascending aorta was resected and a proximal prosthesis-to-prosthesis anastomosis was then performed. Due to complex damage of the innominate artery, arch reconstruction was completed using the 8-mm graft as an extranatomical bypass. The aortic clamp was removed, the myocardium was reperfused, and systemic rewarming accomplished.

Subsequently, CPB was easily weaned off with low dose inotropic support. Operative times were as follows: total CPB time, 200 minutes; aortic cross-clamp, 120 minutes; Kazui's time, 28 minutes.

**Fig. 3:**
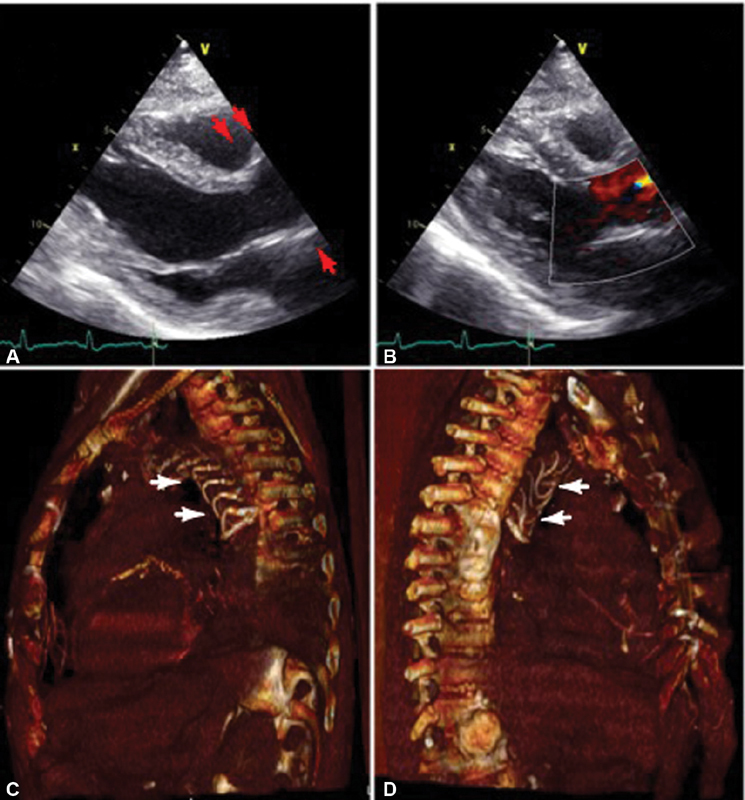
( **A, B** ) Postoperative two-dimensional echocardiogram: parasternal long axis view. ( **A** ) Native aortic valve sparing and reinforcement of the aortic root (shown by red arrows), ( **B** ) color flow mapping, in the parasternal long axis view, shows no aortic regurgitation. ( **C, D** ) Volume rendering of the thorax, left ( **C** ) and right ( **D** ) sagittal views, showing Vascutek Thoraflex hybrid prosthesis in aortic arch and descending aorta. The Vascutek Thoraflex is shown by white arrows.

The postoperative course was uneventful. The patient was neurologically intact. The postoperative CT angiogram scan demonstrated total reconstruction of the aortic arch, patent connection of the branches, and partial thrombosis of the persistent false lumen in the descending thoracic aorta ( Fig. 3 ).

The patient was discharged to a rehabilitation facility after 10 days. At 6-month follow-up, the patient was symptomless. Echocardiogram showed no aortic regurgitation or pericardial effusion ( Fig. 3 ).

## Discussion

This case report demonstrates the contemporaneous use of the Florida sleeve technique plus the Vascutek Thoraflex frozen elephant trunk hybrid prosthesis in an acute Type A aortic dissection.

Acute Type A aortic dissection carries approximately 56% in-hospital mortality when surgical intervention is not performed. 2 Surgical series show a 30-day mortality of 10 to 20%. 3

Different techniques have been applied to treat a widely dissected, aneurysmal aortic root, including the Bentall–De Bono and David operations. 4 The appropriate technique is controversial when there is aortic root involvement and the native aortic valve is normal. New hemodynamic conceptions regarding the importance of avoiding aortic root deformation to optimize transvalvular ejection throughout the cardiac cycle open a new scenario in terms of aortic root replacement or repair. 5

The Florida sleeve operation has been proposed as an alternative method to repair the aortic root and to spare the aortic valve, in the setting of annuloaortic ectasia. Gamba et al 6 demonstrated satisfactory long-term outcome after application of the Florida sleeve technique with a mean clinical follow-up of 34 ± 19 months. The Florida sleeve occasionally has been proposed as a method to treat acute Type A aortic dissection to avoid bleeding and aortic valve replacement. The Florida Sleeve operation raises interest in the treatment of Type A aortic dissection for multiple reasons: (1) it accomplishes aortic valve sparing and annuloplasty; (2) there is no resection of the sinuses of Valsalva; (3) no coronary artery ostial translocation is required; (4) En bloc resuspension of the sinotubular junction to the outer graft is accomplished; (5) external protection of dissected root tissues is achieved without need for reinforcement with Teflon's strips and/or glue; and (6) the adequate sizing of the sinus graft, ensuring the preservation of the functional aortic annulus, could prevent further development of valvular regurgitation.

In the setting of a dissected aortic root, bleeding can be prevented by avoiding suturing through a weakened root wall and avoiding translocation of fragile, dissected coronary, the coronary ostia especially when dissected could be delicate and cumbersome to reattach.

As suggested by the study of Heo et al, 7 the traditional supra commissural graft replacement could be complicated by progressive deformation and dilatation of aortic root requiring surgery; reinforcement of the sinuses of Valsalva, especially with regard to the noncoronary one, choosing an adequate graft could prevents further complication, such as dilation or dissection.

After ascending replacement for Type A aortic dissection, over 70% of patients show persistence of the distal false lumen in late follow-up, and further interventions are frequently needed, either open or endovascular. In the study of Inoue et al, 3 unachieved primary entry resection and absence of total-arch replacement are independent predictors of distal aortic dilatation (≥50 mm) at multivariable analysis.

Our simultaneous use of a frozen elephant trunk (Vascutek Thoraflex hybrid prosthesis) provides many advantages: (1) it permits replacement of the entire aortic arch and supraortic vessels; (2) it secures the isthmic zone, and (3) it creates a thoracic landing zone suitable for subsequent endoprosthesis implantation. 8

In this case report, two specific techniques were used to treat a Type A acute aortic dissection, producing a total proximal aortic replacement. Many positive benefits, including easier and quicker approach in an emergency, derive from the contemporaneous use of these two surgical procedures.

There are many unsolved questions about this hybrid technique, especially for results at long-term follow-up, and further studies are needed, but it could be an easier choice in this peculiar, urgent setting.
